# Positron Emission Tomography/Computed Tomography (PET/CT)-Based, Local Consolidative Radiotherapy after Chemoimmunotherapy in Metastatic, Non-Oncogene-Addicted Non-Small-Cell Lung Cancer (NSCLC)

**DOI:** 10.3390/diagnostics13091538

**Published:** 2023-04-25

**Authors:** Lucia Angelini, Montserrat Pazos, Lukas Käsmann, Farkhad Manapov

**Affiliations:** 1 Department of Experimental and Clinical Biomedical Sciences, University of Florence, 50134 Florence, Italy; 2 Department of Radiation Oncology, University Hospital, LMU Munich, Ziemsenstrasse 1, 80336 Munich, Germany; 3 German Center for Lung Research (DZL), Comprehensive Pneumology Center Munich (CPC-M), 81377 Munich, Germany; 4 German Cancer Consortium (DKTK), Partner Site Munich, 81377 Munich, Germany

**Keywords:** NSCLC 1, multimodal treatment 2, PET/CT 3

## Abstract

The optimal sequence of chemo/immuno- and radiotherapy (RT) in metastatic non-small-cell lung cancer (NSCLC) remains challenging. Here, we describe the case of a 58-year-old female patient with an initially metastasized NSCLC obtaining local and distance durable response after chemo-immunotherapy and local RT associated with immunotherapy maintenance. Our experience offers a valuable perspective in choosing how to combine therapies to ensure the longest possible response in patients with estimated poor prognosis.

A 58-year-old Caucasian woman was referred to our department in February 2022. The diagnosis of NSCLC in the right upper lobe was established in February 2021 (see Figure 1). In particular, she was diagnosed with an adenocarcinoma with G12D mutation in the KRAS gene and PD-L1 0%.

Initial positron emission tomography/computed tomography (PET-CT) staging (March 2021) showed multiple metastases in cervical, mediastinal and paraaortic lymph nodes, pancreatic body, liver and bones resulting in Stage IVB (cT4 cN2 M1c) based on TNM staging (VIII edition). Her general conditions were moderate with an Eastern Co-operative Oncology Group Performance Status of 2. 

Relevant anamnestic findings were smoking until 2016 (40 p/y), pulmonary arteritis in therapy with oral anticoagulant, moderate COPD and hyperlipidemia. 

In May 2021 she started chemo-immunotherapy with Nab-paclitaxel, Carboplatin and Atezolizumab (four cycles), followed by maintenance therapy with Atezolizumab, according to the phase III IMpower130 study (NCT02367781) and recommended by national and international guidelines in first-line treatment of non-squamous, non-oncogene-addicted, metastatic NSCLC [1]. 

In January 2022 at a routine radiological check with CT, despite a good systemic response to treatment, a primary tumor growth with wall thickening of the tumor cavity and a moderate regional lymph node enlargement in the right lung hilum were detected. The PET/CT of February 2022 confirmed a slight increase in metabolic activity only in the tumor cavity, with no activity at mediastinal or cervical level. In a multidisciplinary tumorboard, the recommendation to maintain atezolizumab and carry out thoracic irradiation on right lung and mediastinal lymph nodes was given. 

From February to March 2022 the patient underwent moderately hypofractionated PET/CT-based radiotherapy with volumetric modulated arc therapy with daily image guidance to primary tumor site and right hilum nodes with 42.0/3.0 Gy (see Figure 1). 

Subsequently, the patient was subjected to regular clinical and radiological checks that showed a continuous reduction of the macroscopic tumor burden. 

At the last radiological check in August 2022, PET/CT showed an excellent response according to PERCIST 1.0 with maintenance of the control of distant metastases documented at diagnosis [2]. 

Data from the KEYNOTE-01 trial showed early and long-term interaction between immunotherapy and RT. An updated analysis of the KEYNOTE-001 clinical trial data proposed by Shaverdian et al. found superior progression-free survival (PFS) and overall survival (OS) in patients with a history of irradiation compared to the ones without previous RT (6.3 vs. 2 months and 11.6 vs. 5.3 months, respectively), which shows clearly superior treatment results for patients who previously received RT [3]. There is increasing evidence supporting local ablative treatments (LAT) (surgery or RT) in patients with oligometastatic NSCLC and response to the first-line systemic therapy [4]. 

Gomez et al. in a MDACC multicenter randomized phase II trial showed a significant PFS benefit in oligometastatic NSCLC patients who received aggressive LAT compared to maintenance therapy/observation (14.2 vs. 4.4 months; *p* = 0.022) [5]. 

Furthermore, in the 2019 update, they reported a significant OS benefit (41.2 vs. 17.0 months; *p* = 0.017), without significant differences in toxicity [6]. In addition, Iyengar et al. showed a significant PFS benefit in oligometastatic NSCLC patients who received stereotactic body radiotherapy in addition to maintenance chemotherapy compared to maintenance chemotherapy alone (9.7 vs. 3.5 months; *p* = 0.01) in a smaller monocentric randomized phase II trial [7]. Both studies were closed early to accrual due to the significant PFS benefit seen in the planned interim analysis. Unlike the aforementioned studies, De Ruysscher et al. specifically analyzed a subgroup with brain metastasis treated with LAT, showing that 52% of patients had intracranial progression, but with no in-field recurrences, proving the already known excellent local control given by RT also in the brain in a single-arm phase II trial [8]. 

First progression site during or after chemoimmunotherapy or immune checkpoint inhibition is predominantly the mediastinal lymph node region apart from the brain [9,10]. Therefore, thoracic radiotherapy seems to be logical to increase intrathoracic control and anti-tumor activity due to the recruitment of certain immune cell subsets from peripheral blood [11]. 

Despite several studies investigating oligometastatic settings, there are only a few studies on local consolidative radiation treatment in metastatic NSCLC patients treated with immuno- or chemoimmunotherapy. Most recent findings come from a pooled analysis of 2 phase II randomized trials published by Theelen et al. [12], i.e., MDACC trial [13] and PEMBRO-RT study [14]. The first of these showed that concurrent pembrolizumab and RT was safe and median PFS was significantly improved with the combination of RT and pembrolizumab versus pembrolizumab alone (20.8 vs. 4.6 months, *p* = 0.004) in patients with low PD-L1 expression. The second study showed no significant differences in toxicity and PFS and OS benefit with the addition of radiotherapy to pembrolizumab versus immunotherapy alone. The pooled analysis demonstrated that both PFS (9.0 vs. 4.4 months; *p* = 0.045) and OS (19.2 vs. 8.7 months, *p* = 0.0004) were improved with the addition of RT. Moreover, the addition of RT to pembrolizumab significantly increased the best abscopal response rate compared with pembrolizumab alone (41.7% vs. 19.7%, *p* = 0.0039).

Furthermore, our previous case report described the therapy strategy of multi-site hypofractionated radiotherapy and chemoimmunotherapy for a metastatic pulmonary EBV-associated lymphoepithelioma-like carcinoma [15]. 

Regarding toxicity of the multimodal treatment, a systematic review and meta-analysis including 20 clinical trials and a total of 2027 patients with NSCLC of any tumor stage showed that immunotherapy using PD-1/PD-L1 inhibitors and RT did not increase the serious adverse event rates (≥grade 3) compared with non-combination therapy [16]. 

In a pilot study on metastatic NSCLC, Qin et al. showed that hypofractionated image-guided radiation therapy plus atezolizumab resulted in an overall response rate of 25% and disease control rate of 50%, with similar incidence of grade 3 adverse events compared with patients who received atezolizumab alone. These results, although suffering from severe limitations including the small sample size, encourage further investigation [17]. 

In summary, available data suggest the efficacy and safety of combining immunotherapy and RT; however, their systematic integration in clinical practice remains to be explored. The results of several ongoing studies are warranted, e.g., ALLIANCE A08002 [18] and ESPERA trial [19]. The first is a randomized phase II/III trial based on the results of PEMBRO-RT study [14] evaluating the anti-tumor activity of systemic immuno- or chemo-immunotherapy in patients with stage IV PD-L1 negative NSCLC. The second is a randomized phase II trial which is evaluating the efficacy and safety of adding RT to pembrolizumab-pemetrexed maintenance in advanced NSCLC patients experiencing disease response or stability after chemo-immunotherapy induction. 

Our report questions the role of local consolidative treatment after chemoimmunotherapy in metastatic NSCLC. Current literature shows no solid data and, therefore, we describe a valuable perspective in choosing how to combine therapies to ensure the longest possible response in NSCLC patients with estimated poor prognosis.

## Figures and Tables

**Figure 1:**
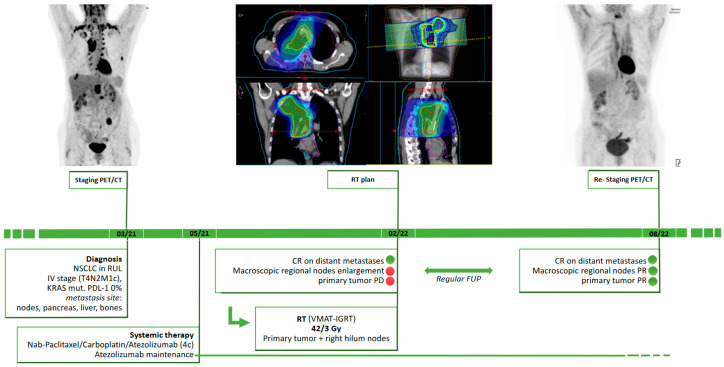
Timeline of patient’s treatment and follow-up. Abbreviations: CR: complete remission, FUP: follow-up program, KRAS: Kirsten rat sarcoma virus, NSCLC: non-small-cell lung cancer, RUL: right upper lobe, PR: partial remission, PD: progressive disease, PET/CT: positron emission tomography/computed tomography, VMAT-IGRT: volumetric modulated arc therapy with daily image guidance.

## Data Availability

The data presented in this study are available in Figure 1.
